# *Aspergillus fumigatus* mitogenomes and their influence on azole-resistant and -susceptible populations

**DOI:** 10.1038/s44259-025-00083-6

**Published:** 2025-02-27

**Authors:** Endrews Delbaje, Laís Pontes, Johanna Rhodes, Jacob Steenwyk, Ling Lu, Thaila F. dos Reis, Antonis Rokas, Gustavo H. Goldman

**Affiliations:** 1 Faculdade de Ciências Farmacêuticas de Ribeirão Preto, Universidade de São Paulo, Ribeirão Preto, Brazil; 2 Department of Medical Microbiology, Radboud University Medical Center, Nijmegen, The Netherlands; 3 Howards Hughes Medical Institute and the Department of Molecular and Cell Biology, University of California, Berkeley, Berkeley, California USA; 4 Jiangsu Key Laboratory for Microbes and Functional Genomics, Jiangsu Engineering and Technology Research Centre for Microbiology, College of Life Sciences, Nanjing Normal University, Nanjing, China; 5 Department of Biological Sciences and Evolutionary Studies Initiative, Vanderbilt University, Nashville, Tennessee USA; 6 National Institute of Science and Technology in Human Pathogenic Fungi, São Paulo, Brazil

**Keywords:** Genetics, Microbiology, Molecular biology

## Abstract

The role of the fungal mitochondria goes far beyond energy metabolism. The genomes of 318 *Aspergillus fumigatus* clinical and environmental isolates from different geographic origins were analyzed aiming to study the mitochondrial sequences from populations sensitive and resistant to azoles. Our results show that *A. fumigatus* mitogenomic sequences are very conserved and only show variation in small intergenic regions and one intronic sequence in the *cox*3 gene. Furthermore, a genome-wide association analysis of accessory mitochondrial genes revealed potential mitochondria-based genotypes that may interact synergistically with the ergosterol biosynthesis pathway to confer the resistant phenotype. This includes a mutation in the AMID-like mitochondrial oxidoreductase (*aifA*, AFUA_3G01290) and the absence of the mitochondrial carrier protein (*pet8*, AFUA_8G01400). Deletion of these genes did not change the azole-susceptibility but increased the azole-persistence, suggesting mitochondrial genes could be involved in azole-persistence. Our work opens new hypotheses for the involvement of mitochondria in *A. fumigatus* azole-resistance.

## Introduction

*Aspergillus fumigatus* is a saprophytic fungus ubiquitously distributed in the environment with worldwide distribution^1^. In humans it can be an opportunistic pathogen and it is the most predominant *Aspergillus* spp. causing Invasive Pulmonary Aspergillosis (IPA) in immunosuppressed patients^2^. Interest in IPA has recently increased due to high mortality rates among patients co-infected with SARS-CoV-2, the virus that causes COVID-19^3^. Recently, *A. fumigatus* was included in the World Health Organization’s (WHO) inaugural list of priority fungal pathogens^4^. The primary treatment against *A. fumigatus* infection is the administration of azole antifungal agents^5^, which target the Cyp51A protein in the ergosterol biosynthesis pathway^6^. Among humans infected with invasive forms of aspergillosis, azoles—such as voriconazole, isavuconazole, itraconazole and posaconazole—are the frontline clinical defense^7,8^. However, a globally escalating issue is the increasing frequency of infections with strains resistant to this class of antifungal agents^9^, and in some cases, the acquisition of resistance to azoles can occur during the infection treatment^10^. Furthermore, the evolution of resistance can evolve in the environment due to the extensive use of azoles like benzimidazoles against plant fungal pathogens^11,12^, which can persist in the environment for a long period due to the ability to resist changes in its molecular structure^13^. Other frequent sources of azole exposure are the contamination of soil, wastewater, and sewage^14^.

The primary mechanism of azole resistance in *A. fumigatus* is attributed to point mutations, characterized by non-synonymous nucleotide substitutions in the *cyp51A* gene, and tandem-repeat (TR) mutations occurring in the promoter region^15^. However, other resistance mechanisms are known, such as genes encoding efflux pumps, including ABC and MFS transporters, which are the main mechanism in eukaryotic organisms for removing toxins from the cell^16^, and their overexpression can lead to azole resistance in *A. fumigatus*^17^. Recently, non-*cyp51A* mediated resistance in *A. fumigatus* isolates has garnered increased interest. Current evidence implies the presence of numerous uninvestigated mutations that influence cellular processes that lead to resistance^18–20^. Among these processes, the impact of mitochondria on the virulence and resistance of medically important fungi has become increasingly evident^21,22^.

Previous research has shown that mitochondrial dysfunction can lead to azole resistance. The *cox10* mutants, characterized by defective heme A biosynthesis, have been observed to upregulate calcium signaling genes. This upregulation results in the accumulation of cytosolic Ca^2+^ and the nuclear translocation of the transcription factor CrzA^22^. In another study, *A. fumigatus* null mutants in three genes that control mitochondrial morphology, *fis1* (encoding a mitochondrial outer membrane protein), *mdv1* (encoding a WD repeat-containing protein) and *dnm1* (encoding a dynamin-like GTPase), showed increased resistance to azoles in conjunction with reduced growth and aberrant mitochondrial morphology^23^. Bcs1A of *A. fumigatus* was found to modulate the azole response via efflux pump expression, where its null mutant shows increase in resistance^24^. In addition to these genes, *fzo1* and *oxa1*, encoding a mitochondrial protein required for the assembly of the cytochrome c oxidase complex and the F0 ATPase sub-complex, respectively, which impact mitochondrial morphology, have also been associated with the regulation of the gene *pdr* (pleiotropic drug resistance) expression in *S. cerevisae*, leading to increased drug resistance. Notably, the corresponding *A. fumigatus* orthologs remain uncharacterized^21^. Another process associated with resistance is related to the communication between the nucleus and mitochondria. In a study on mitochondrial dysfunction in *S. cerevisiae*, it was observed that Rtg1p and Rtg2p, responsible for signaling between the nucleus and mitochondria, including organelle dysfunction events, can control the activation of *pdr* genes^21^. In *A. fumigatus*, an Rgt2 homolog (AFUA_8G00730) exists, but its potential association with resistance remains unexplored.

These experimental investigations into mitochondria underscore the potential relationship between the mitochondrial genes of *A. fumigatus* and the evolution of azole resistance. Despite the existing evidence, there is a lack of detailed genomic studies examining the relationship with azole resistance and its potential impact on mitochondrial genome organization, as well as mitochondrial encoded (ME) and nuclear-encoded mitochondrial (NEM) proteins. Furthermore, the degree of intraspecific variation in *A. fumigatus* ME and NEM genes, which could provide valuable insights into the evolution of the species, remains unexplored. In the present study, mitochondrial sequences derived from *A. fumigatus* populations across various countries, encompassing both azole-sensitive and -resistant isolates (*Cyp51A*-dependent and –independent), were examined for intraspecies variation. Furthermore, an analysis was conducted to ascertain the associations of specific mutations with azole resistance.

## Results

### Characterization of *A. fumigatus* mitogenomes and nuclear-encoded mitochondrial protein genes

A total of 318 genomes and mitogenomes from both azole-resistant and -susceptible *A. fumigatus* isolates were assembled from whole genome sequencing (WGS) data. BUSCO analysis showed an average genome completeness of 96.34% (range: 92.9% to 96.6%; Supplementary Data 2), with an average of fragmented BUSCOs of 1.15% ranging from 1% to 3.1%. From the 318 WGS data, 316 mitogenomes were assembled that resulted in a singular circular contig, with sizes that ranged from 30693 to 31803 bp and showed low variation in GC content (25.33–25.49%; Supplementary Data 2). The mitogenome synteny showed only two general structures, with 286 mitogenomes (90.51%) with the same sequence structure with sizes ranging from 31680 bp to 31803 bp and 30 mitogenomes (9.49%) with an alternative structure with sizes from 30693 bp to 30757 bp. Two mitogenomes (isolates VPCI_1882 and VPCI_469) could not generate a good quality mitogenome assembly due to low genomic coverage and were discarded from the analysis.

**Fig. 1:**
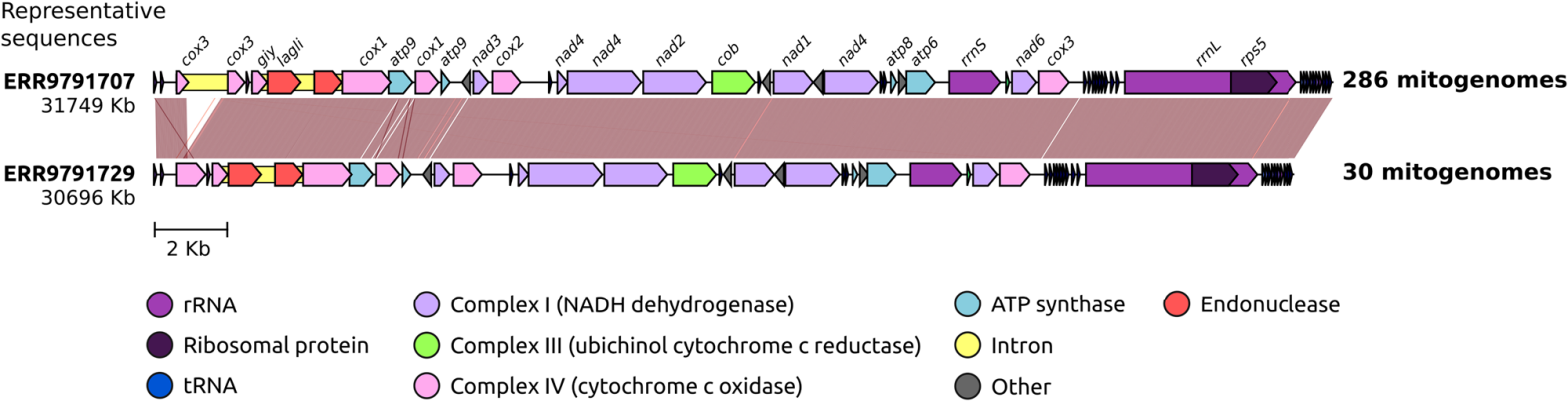
Genetic structure of 318 *A. fumigatus* mitogenomes. Syntenic comparison of two structural representative mitogenomes. The number of mitogenome sequences with identical structure to the representatives is indicated.

The main difference between the structural groups was the presence of intronic sequences in the gene encoding cytochrome c oxidase (*cox3* upstream of *cox1*) (Fig. 1). In all the mitogenomes examined, intronic sequences were observed within the cytochrome c oxidase I (*cox1*) gene. These sequences are associated with two types of homing endonucleases, namely GIY-YIG and LAGLIDADG, which exhibited minimal variation. Specifically, the 316 GIY-YIG endonuclease sequences were found to be identical for all samples. Across all mitogenomes, 27 transfer RNA (tRNA) sequences remained consistent in localization and classification of tRNA. They were classified into 22 distinct tRNA types, with the most frequently occurring type being methionine tRNA (trnM) with three copies in each isolate. The pan-mitogenome showed 19 core genes and 2 accessory genes, the latter comprising two variants of the *cox3* gene upstream of *cox1* gene and one extra smaller *atp9* gene in 60% of isolates (Supplementary Data 3). The variation of *cox3* gene is correlated to the intronic sequence variation, where all mitogenomes with the extra intron showed variation in this gene. There was no observed correlation of intronic variation and resistance phenotype.

**Fig. 2:**
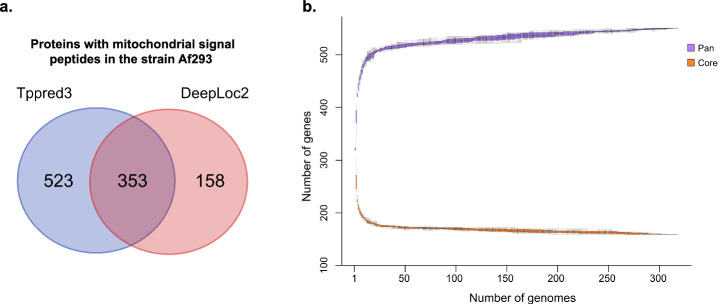
Nuclear-encoded mitochondrial (NEM) orthologs of 318 *A. fumigatus* genomes. **a** Venn diagram depicting the proteins with peptide signal for mitochondrial transport found by each program used. **b** Gene content plot showing the accumulated gene set and core genes based on putative nuclear-encoded mitochondrial proteins.

To identify NEM proteins, two distinct signal peptide prediction tools to detect mitochondrial localization were employed. The intersection of results from Tppred3 and DeepLoc2 in the reference strain Af293 identified 353 proteins with putative mitochondrial signal peptides (Fig. 2a). This set was used as a reference list of NEM proteins for subsequent analysis. Additionally, it included previously characterized mitochondrial proteins from Af293 that were not predicted to contain signal peptides by the combination of both programs. This resulted in a final reference list with 468 NEM proteins from Af293 (Supplementary Data 5).

Across the 318 studied genomes, the intersected results generated by these predictors revealed an average of 321 proteins exhibiting mitochondrial localization, ranging from 313 to 328 proteins for each isolate. The NEM orthologs revealed a core set comprising 164 genes, an accessory collection with 359 genes and 27 singleton genes. This resulted in a total of 550 NEM orthologous genes (Fig. 2b). According to the calculated Heap’s law model, with an alpha value of 2, the saturation for discovery of new NEM orthologs is almost reached.

**Fig. 3:**
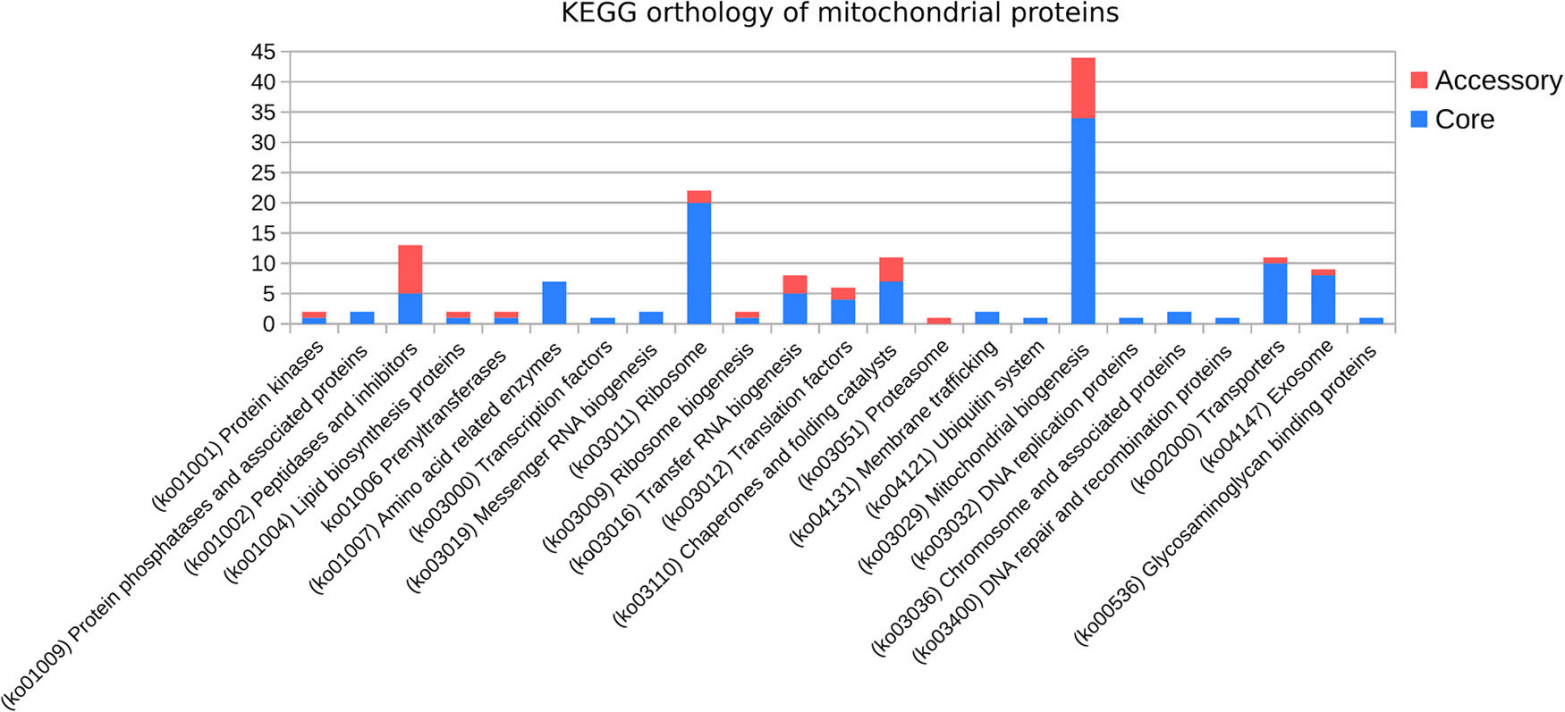
KEGG categorization of core and accessory mitochondrial genes.

The functional categorization of the core and accessory putative NEM proteins, based on KEGG (Fig. 3), revealed that most of the identified protein categories are associated with Mitochondrial biogenesis (44 KEGG), Ribosomes (22 KEGG), Peptidases and inhibitors (13 KEGG), Chaperones (11 KEGG), and Transporters (11 KEGG) (Fig. 3). The accessory genes were predominantly distributed across the categories of Peptidases and inhibitors, tRNA biogenesis, Mitochondrial biogenesis, Chaperones, and Translation factors (Fig. 3). Within the category of Mitochondrial biogenesis, the accessory genes were found to be orthologous to mitochondrial quality control factors, such as the iron-sulfur cluster assembly 2 protein (K22072), Fe-S cluster biogenesis protein (K22073), and respiratory chain complex assembly proteins COX11 (K02258), COX15 (K02259) and SURF1 (K14998). Additionally, orthologs of the Mitochondrial protein import machinery were identified, which include the calcium uniporter protein (K20858) and the mitochondrial protein import protein ZIM17 (K17808).

### Mitochondrial proteins and azole resistance

**Fig. 4:**
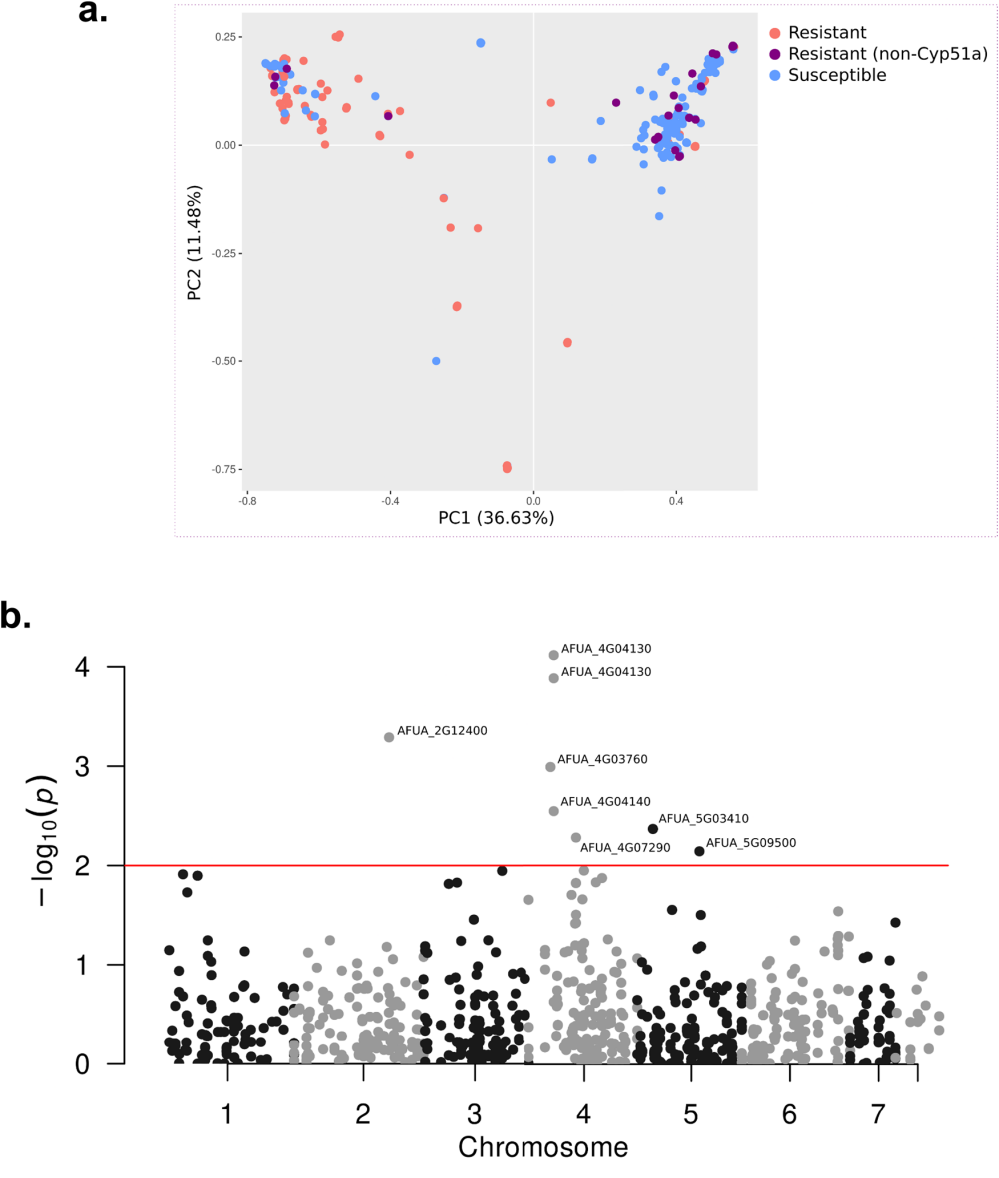
GWA analysis based on SNPs in nuclear encoded mitochondrial genes. **a** PCA biplot using all mitochondrial SNPs comparing azole-resistant and -susceptible isolates, and isolates with *Cyp51A*-independent azole resistance. **b** Manhattan plot of SNPs significantly associated with the resistance phenotype. Red line depicts the *p*-value cutoff for significance.

Variant calling using the reference of *A. fumigatus* Af293 found 3781 SNPs distributed across 468 mitochondrial genes were filtered to retain only those variants present in a minimum of 5% of the samples. This resulted in a refined set of 1749 SNPs spanning 417 mitochondrial genes. Principal Component Analysis (PCA) based on these SNPs revealed a partial clustering among the phenotypes. Although a clear distinction was observed, there was an overlap involving a subset of samples (Fig. 4a).

GWA was performed to find possible associations of azole resistance with SNPs localized in mitochondrial genes. The analysis found no association for genes in the mitogenome. For genes that synthesize mitochondrial nuclear-encoded proteins, the analysis showed significant association for ten variants in seven genes (Fig. 4b), where three of them showed non-synonymous variants (Supplementary Data 4): a mitochondrial ATPase (AFUA_4G04130), an ATP synthase involved in oligomycin sensitivity (AFUA_2G12400) and an exopolyphosphatase (AFUA_5G03410).

A GWA study was conducted to identify associations between resistance and key components involved in mitochondrial fusion and fission in *A. fumigatus*. These components were analyzed separately due to the absence of mitochondrial transport peptide signals in some genes, specifically: *mdv1* (AFUA_5G13140), *dnm1* (AFUA_8G02840), Rtg2p_1 (AFUA_6G08350A), and Rtg2p_2 (AFUA_8G00730A). Other components, encoded by *fis1* (AFUA_2G13320) and *oxa1* (AFUA_5G03640), which possess mitochondrial signal peptides, were included in previous analyses. This selection is based on the review by Neubauer and colleagues^23^. A statistically significant association was identified using a chi-squared analysis. A SNP in *fzo1* (chi-squared test, *P* < 0.0001, d.f. = 1), located within an intronic sequence, was found in 10 resistant isolates (9 *cyp51A*-independent and 1 *cyp51A* mutant) and 2 susceptible isolates. Additionally, a significant association was observed in *fis1* (chi-squared test, *P* < 0.0001, d.f. = 1), present in 67 resistant isolates (4 *cyp51A*-independent and 63 *cyp51A* mutants) and 42 susceptible isolates.

**Fig. 5:**
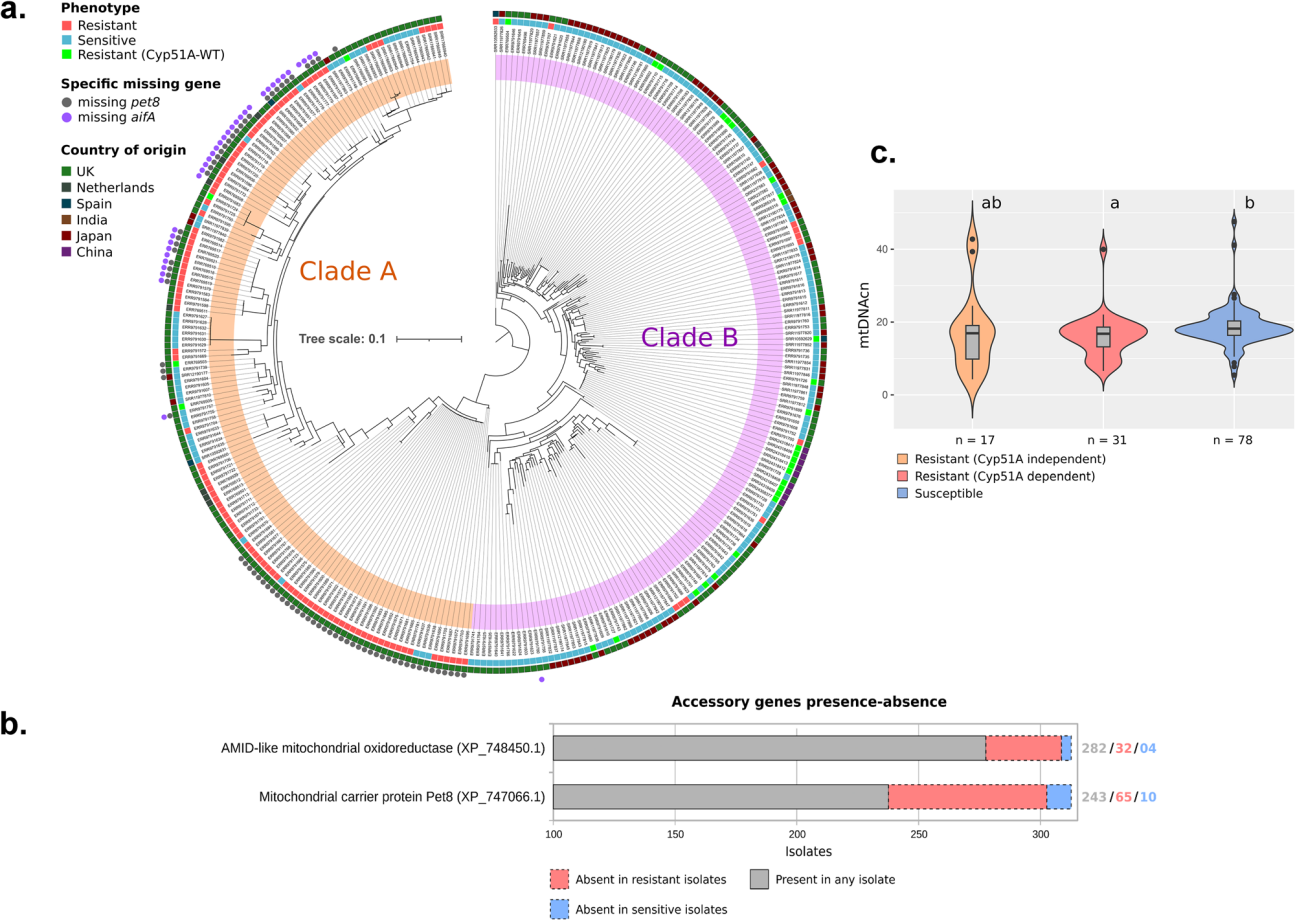
Population comparison based on azole-resistance. **a** Phylogenetic tree with bootstrap support based on SNPs in nuclear encoded mitochondrial genes. Resistance phenotype, Cyp51A genotype and country of origin are highlighted by coloured boxes and the absence of the genes pet8 and aifA are indicated by coloured circles. Clades A and B are highlighted by orange and pink bands, respectively. Tree scale represents nucleotide substitution per site. **b** Accessory genes significantly associated with resistance identified by Scoary and their occurrence according to phenotype. **c** Violin-plot and boxplot of mitochondrial DNA copy number comparing resistant and susceptible isolates, with *p*-value indicated for the Pairwise comparisons using Wilcoxon rank sum exact test and Bonferroni adjustment method.

To visualize the evolutionary relationship among isolates based on nuclear-encoded mitochondrial proteins, a phylogeny based on these gene variants was constructed (Fig. 5a). The phylogenetic tree showed the composition of two clades, named A (*n* = 150) and B (*n* = 168), following the same nomenclature of Sewell and colleagues^25^. Clade A was mainly composed of resistant isolates, representing 72% of the clade (108/150), where 105 isolates have a *cyp51A* mutation that has been previously associated with resistance, based on the MARDy database v1.1 for resistance variants^26^. In contrast, Clade B isolates were mostly azole-susceptible, with only 40 azole-resistant (23.8%), from which 28 do not show mutations in the gene *cyp51A* associated with antifungal resistance.

To investigate the evolution of mismatch repair mechanism variations in *A. fumigatus* in relation to azole resistance and its distribution across subpopulations, we examined the msh6 gene (AFUA_4G08300) for mutations. A significant proportion of isolates within clade A (86%; 129/150) harbored the non-synonymous SNP G698C, resulting in an amino acid substitution (G233A). In contrast, only 1.78% (3/168) of isolates in clade B exhibited this variant, indicating a strong association with the mutation (chi-squared test, *P* < 0.0001, d.f. = 1).

Discriminant analysis of principal components (DAPC) subdivided the populations in four groups (Supplementary Figs. 2 and 3) with two groups in the clade A and two in the clade B. From the two groups in clade A, one is notably composed of azole-resistant isolates (35 resistant isolates out of 39 isolates), with all isolates from different regions of the United Kingdom (UK). Interestingly, all these isolates lack the gene encoding *Pet8*. The other subgroup in clade A is geographically more heterogeneous, with isolates from the UK, Netherlands, Spain, Japan, and India, comprising^27^ azole-resistant isolates out of 108. A subset of 30 isolates from this group lacks a functional *aifA* and *pet8*. Among these, 27 isolates are azole resistant. Unlike the isolates that only lack *pet8*, this subset includes isolates from geographically distant origins worldwide, including Spain, UK, Netherlands, and India. As for the two subgroups identified within clade B, they display geographical heterogeneity and are predominantly composed of azole-susceptible isolates. A significant proportion (chi-squared test, *P* < 0.0001, d.f. = 1) of the wildtype *cyp51A* resistant isolates are distributed across these two groups.

A correlation analysis was conducted with Scoary^28^, considering both azole-resistant and -sensitive phenotypes in relation with the presence or absence of genes in the isolates. The analysis indicated a correlation between the resistance phenotype and the absence of genes encoding the mitochondrial carrier protein (*pet8*, AFUA_8G01400A) and AMID-like mitochondrial oxidoreductase (*aifA*, AFUA_3G01290) (Fig. 5b and Supplementary Data 6). It was observed that out of 75 isolates lacking the *pet8*, 65 (86.7%) exhibited resistance. Similarly, among the 35 isolates devoid of *aifA*, 31 (88.6%) were resistant, with both genotypes representing the highest discrepancy in gene absence in the resistant phenotype, when compared with susceptible isolates. Notably, there was a considerable overlap of both genotypes, with both genes absent in 30 of the isolates (Fig. 5a), representing a significant association (chi-squared test, *P* < 0.0001, d.f. = 1). Furthermore, isolates missing both genes shared a closely related phylogenetic pattern of SNPs. The phylogenetic tree, constructed based on 1749 SNPs, revealed two distinct subclades within clade A. One subclade predominantly consisted of isolates lacking both genes, while the other primarily included isolates missing only the AMID-like oxidoreductase gene (Fig. 5a), being congruent with the subclade division found by DAPC analysis (Supplementary Fig. 2). In total, 25 genes showed significant association with the resistance phenotype, accounting for cyp51A dependent and independent resistance.

The average fixation index (F_ST_) was calculated for populations grouped by phenotype to infer differentiation among these populations based on SNPs. This was achieved by measuring the difference within populations against the difference between populations, where an F_st_ value of 0 indicates no differentiation and an F_ST_ value of 1 indicates total differentiation^29^. The highest average F_ST_ (0.144) was observed between resistant *cyp51A* mutants and resistant *cyp51A*-independent populations, with the peak F_ST_ level located on chromosome IV near the *cyp51A* gene (Supplementary Fig. 5 and Supplementary Data 7). The lowest average F_ST_ (0.016) was found between susceptible and resistant *cyp51A*-independent populations, indicating no clear population distinction between these phenotypes. This lack of distinction is corroborated by DAPC analysis, which shows no clear clustering within clade B based on these phenotypes.

Tajima’s *D* was calculated for each phenotype, to infer the difference between the expected and the observed variation of each population based on SNPs, where a positive value indicates that the observed variation is higher than the observed and a negative value for observed variation lower than the expected. The average Tajima’s *D* for resistant *cyp51A*-independent isolates was −0.01 (Supplementary Data 7), suggesting that the observed variation aligns with the expected variation for a population under no selection. In contrast, the susceptible and resistant *cyp51A* mutant populations had average Tajima’s *D* values of −0.56 and 0.31, respectively, indicating different selection patterns among the three populations (Supplementary Data 7).

The mitochondrial DNA copy number (mtDNAcn) was evaluated, inferred from the average coverage of genomes and mitogenomes from genomes with high coverage data (above 50x), to determine potential variations in mitochondrial quantity within cells relative to the phenotype. The results showed a significantly lower copy number in the population of resistant (Cyp51A dependent) isolates, determined by the Wilcoxon rank sum exact test (*p* < 0.05) in comparison to susceptible isolates. In addition, these resistant isolates exhibited less variation in mtDNAcn compared to Cyp51A independent resistant isolates, as evidenced by their genome coverage (Fig. 5c). These findings show that there is a distinct genetic background involving mitochondrial adaptations between susceptible and resistant isolates, which could be determined also by the type of resistance, which are dependent on the phylogenetic affiliations of isolates with clade A, clade B and its subclades.

### Construction of deletion mutants for the genes encoding the mitochondrial carrier protein (*pet8*, AFUA_8G01400A) and AMID-like mitochondrial oxidoreductase (*aifA*, AFUA_3G01290)

**Fig. 6:**
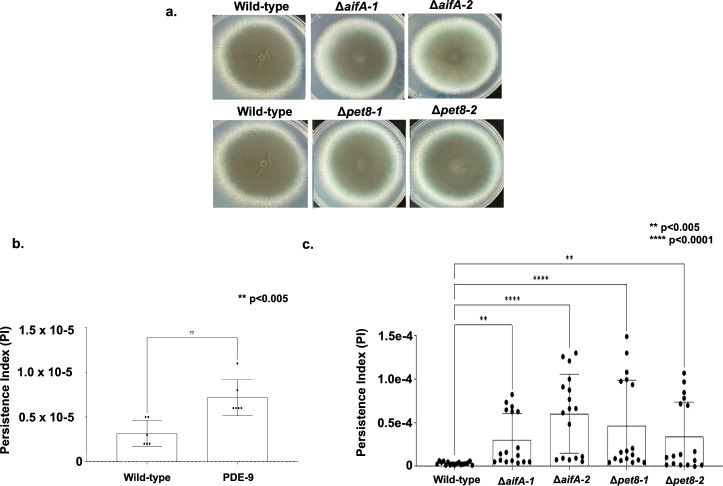
Deletion analysis of *A. fumigatus aifA* and *pet8.* **a** Radial growth and morphology of the corresponding A1160 wild-type and two independent null mutants for *aifA* and *pet8*. **b**, **c** Voriconazole persistence index for *A. fumigatus* wild-type, PDE-9 and two independent null mutants for *aifA* and *pet8*.

We constructed two independent mutants for *A. fumigatus pet8* and *aifA* in the background of the A1163 *pyrG*^*-*^ strain aiming to verify a possible role in azole resistance. We have used these two independent mutants to confirm the observed phenotypic effects were not due to the occurrence of possible secondary mutations caused by the genetic transformantion procedure. These mutants showed comparable growth phenotypes to the wild-type strain (A1160 *pyrG*^+^ strain; Fig. 6a), and most importantly their Minimal Inhibitory Concentrations (MICs) for posaconazole (MIC = 0.015 µg/ml), voriconazole (MIC = 0.5 µg/ml), itraconazole (MIC = 1.0 µg/ml), isavuconazole (MIC = 1.0 µg/ml), amphotericin (MIC = 1.0 µg/ml) and caspofungin (minimal effective concentration, MEC = 0.5 µg/ml) were the same than the wild-type strain, suggesting these mutations are not impacting the azole-resistance in the A1163 background.

### *A. fumigatus pet8* and *aifA* null mutants have increased azole-persistence

To check if the deletion of *pet8* or *aifA* could be involved in the azole persistence, we developed a quantitative assay that uses supra-MIC concentrations (4xMIC) of azoles combined with extended exposure of the strains (96 h at 37 ^o^C) to the azole and subsequent plating to evaluate the number of survival colony forming units (CFUs). Persistence index (PI) refers to the number of CFUs observed after exposure of fungal conidia to 4x MIC of voriconazole divided by the initial number of conidia used in the assay. To validade this assay, we first determined the azole persistence of A1160 parental strain and a previously described high persister strain, PDE-9. As expected the PDE-9 strain has a higher PI than A1160 (PI = 6.9e^6^, about twice more azole persistence; Fig. 6b). The Δ*pet8* and Δ*aifA* mutants showed a higher PI of about 3.0e^−5^ and 4.0e^−5^, respectively, when compared to the wild-type strain A1160 (PI = 3.39e^−06^; Fig. 6b), suggesting these genes could be involved in azole-persistence, and surprisingly they are more azole persistent than the PDE-9 strain. It is important to notice that the voriconazole MICs of about ten persisting colonies derived from the clinical wild-type, PDE-9 and the mutant strains have the same MIC from the parental strains.

## Discussion

This study showed a comprehensive characterization and comparison of an unprecedented number of *A. fumigatus* mitogenomes. The sample set included both environmental and clinical azole-resistant and -susceptible representatives from at least six distinct global regions: UK, Ireland, Japan, India, China, and the Netherlands, in addition to four isolates of unknown geographical origins (Supplementary Data 1). The genomic data employed in this study provided sufficient coverage, facilitating the assembly of hundreds of high-quality nuclear genomes and closed circular mitogenomes. The analysis reveals minimal variation among the mitogenomic sequences of *A. fumigatus* isolates, with limited differences observed primarily in the presence of introns and intergenic regions. These findings are consistent with the observations of a previous study by Joardar and colleagues^30^, which examined interspecific mitogenome variations, however, there were discrepancies in the annotation, where our results show an additional *cox3* gene with intronic sequence (Fig. 1), whereas the previous work^30^ annotated the gene as hypothetical. This can probably be explained by the development of new tools and more enriched databases that are currently available. Here, it is further established that within a large population of *A. fumigatus*, the mitogenome sequences are highly conserved. Moreover, it was found no correlation between SNPs in the mitogenome sequences and azole resistance phenotype.

Through signal peptide prediction analysis, we observed novel nuclear proteins that are potentially transported to the mitochondria of *A. fumigatus*, identifying a total of 468 nuclear-encoded mitochondrial proteins. In comparison, the reference strain *A. fumigatus* Af293, has 150 proteins encoded in its genome that are currently annotated as ‘mitochondrial’ (assembly accession GCA_000002655.1) and many of these annotations are putative, based on homology with other species. Additionally, the association analysis of the azole-resistance phenotype with these mitochondrial proteins, based on gene SNPs, revealed a correlation with the phylogenetic composition of the isolates, where a clear clustering of the resistant isolates was observed in the phylogenetic analysis and in the PCA analysis. A similar phylogenetic pattern was observed by Sewell and colleagues^25^ and Rhodes and colleagues^31^, based on variant calls of whole genomes, where the isolates also clustered in two groups, A and B, with the former group being highly enriched for azole resistant isolates. DAPC analysis further subdivided the population into four clusters, and interestingly, they are not explained mainly by geographical origin, with the exception of cluster 2 from clade A, composed only with isolates from the UK.

The Genome-Wide Association (GWA) analysis revealed three associations of statistical significance. Among these, two genes, namely the ATP synthase involved in oligomycin sensitivity (AFUA_2G12400) and an exopolyphosphatase (AFUA_5G03410), have not been previously correlated with azole sensitivity in existing literature. Conversely, the third found in the mitochondrial ATPase gene (*afg1*, AFUA_4G04130), has been reported to exhibit negative expression in azole resistant *Cryptococcus neoformans*^32^. The observed correlation between the absence of the genes *pet8* (AFUA_8G01400A) and *aifA* (AFUA_3G01290) with the azole-resistance phenotype in *A. fumigatus* suggests a potential mechanism of resistance enhancement. This mechanism might be attributed to the absence or compromised functionality of these specific mitochondrial genes. The AMID-like oxidoreductase *aifA* gene in *A. fumigatus* is homologous to the apoptosis-inducing factor (AIFM2) in *Saccharomyces cerevisiae*^33^. Notably, AFUA_3G01290 has been observed to be upregulated in the presence of quinoline bromoquinol, potentially inducing apoptosis in *A. fumigatus* through oxidative stress^33^. Pet8 has a yeast homolog known as SAM5. SAM5 is an S-Adenosylmethionine (SAM) transporter involved in methyl group transfers. In eukaryotes, SAM5 can regulate the methylation of DNA, tRNA, and rRNA. The absence of this gene in yeast has been linked to growth defects due to mitochondrial malfunction^34^. The concurrent absence of the Pet8 and AMID-like oxidoreductase genes in azole-resistant isolates could suggest a potential synergistic mechanism of resistance. This mechanism might involve a malformation, possibly due to the lack of methylation regulation by Pet8, leading to apoptosis. However, this apoptosis could be inhibited due to the absence of the AMID-like oxidoreductase gene. Therefore, the absence of this apoptosis inducer, coupled with a mutation causing mitochondrial dysfunction and azole resistance, could constitute a significant synergistic mechanism of resistance. We deleted both genes in the reference *A. fumigatus* strain A1163 and both deletion mutants have comparable azole MIC values to the corresponding wild-type strain. These results suggest both mutations are dependent on the genomic architecture of the azole-resistant strains.

Bacterial persistence has been defined as the ability of a sub-population of a bacterial population to survive to supra-MIC concentrations of a specific drug without changing their original MICs to this drug^35,36^. However, little is known about drug persistence in filamentous fungi. Recently, we described *A. fumigatus* is able to show voriconazole-persistence but the mechanism of action of this persistence remains to be determined^37–39^. Here, we investigated if Pet8 and AifA are involved in vorizonazole-persistence. Surprisingly, both null mutants for the genes encoding these proteins showed about tenfold more vorizonazole-persistence than the wild-type strain, suggesting mitochondrial genes could be involved in azole-persistence. It is possible to hypothesize that azole-persistence could precede azole-resistance allowing the survivors time for evolving drug resistance. However, it remains to be determined how these genes are impacting azole-persistence and how they contribute to the evolution of azole-resistance.

Functional annotation of mitochondrial proteins, based on KEGG, has identified the Mitochondrial Calcium Uniporter (MCU) as an accessory protein. MCU plays a crucial role in mediating Ca^2+^ transport within the mitochondria and typically forms a unit with MICU1, a configuration that is generally conserved across at least 247 eukaryotes, being widespread in Plantae and Metazoa, but with a limited presence in Fungi^40^. Interestingly, *A. fumigatus* possesses the MCU ortholog AFUA_4G10310 but lacks MICU1. This condition, also observed in a few other fungal species such as *Neurospora crassa*, leads to the hypothesis that MCU may have lost its Ca^2+^ uptake ability in these organisms^40^. The observation that this gene is part of a non-core set in *A. fumigatus* aligns with the assumption that it may have evolved to perform a different function, potentially acquiring a new, non-essential role. Here, we found no correlation in the presence/absence of the gene with azole resistance, with this gene present in 33 azole-resistant and in 23 azole-susceptible isolates. However, previous work by Li and colleagues^22^ showed a connection of MCU with resistance, where it was identified up-regulation of the gene MCU in the multidrug resistant *cox10* mutant. This acquired resistance was related to the activation of calcium signaling and increased expression of drug efflux pumps.

Azole-resistant isolates (Cyp51A dependent) were inferred to possess a reduced number of copies of mitochondrial genomes. Resistant Cyp51A independent isolates showed more variation and no significant difference, but this could be a result of the considerable lower number of representant isolates, impairing a robust statistical comparison for these isolates. Literature reports associating mitochondrial copy number with phenotypic variations in fungi are limited. However, one study demonstrated a reduction in mitochondrial copy number in calcineurin Δ*calA* mutant strain^41^. From this observation, it could be hypothesized that these isolates may exhibit a decreased metabolic rate. This hypothesis is supported by evidence suggesting that cells with an increased mitochondrial content can exhibit enhanced oxidative phosphorylation activity^42^. Furthermore, it has been reported that *A. fumigatus* strains which have acquired resistance in patients exhibit a reduced growth rate as a fitness cost^43,44^. This observation aligns with the hypothesis and warrants further investigation into the potential link between mitochondrial copy number and azole resistance.

Most of the *cyp51A*-independent resistant isolates (92.8%) are found within clade B of *A. fumigatus*, which is predominantly composed of azole-sensitive isolates (76.2%). These isolates do not exhibit phylogenetic clustering, indicating multiple de novo origins of *cyp51a*-independent resistance mechanism(s). This observation suggests that *cyp51A*-dependent resistance mutations are more likely to emerge in clade A strains, possibly due to a more conducive genetic background. A genome-wide analysis of clade A and B isolates conducted by Rhodes and colleagues^31^ revealed that *cyp51A*-dependent azole-resistant isolates exhibit signatures of positive selection across multiple genomic regions, suggesting a polygenic basis for the resistance phenotype. It is plausible that clade B possesses a genetic background that is less conducive to the development of *cyp51A*-based resistance, and this could account for the observed higher prevalence of alternative resistance mechanisms within this clade.

It has been suggested that the multi-azole resistance observed in clade A is due to an increased propensity for resistance development, attributed to modifications in the DNA mismatch repair system. This is evidenced by a variant in the msh6 gene (G698C) in *A. fumigatus*, which occurs almost exclusively and dominantly in clade A. The *MSH6* gene is part of the MutSα complex, responsible for recognizing base mismatches and small loops^45,46^. Additionally, Tajima’s D results indicate distinct selection patterns for mitochondrial genes in both resistant cyp51A mutants and resistant cyp51A-independent populations, with resistant cyp51A mutants experiencing greater selective pressure.

In summary, this study has explored potential mechanisms underlying azole resistance in *A. fumigatus*. The correlation between the absence of specific mitochondrial genes and azole resistance suggests a complex interplay of genetic background and metabolic factors contributing to resistance associated with the ergosterol synthesis pathway. The observed reduction in mitochondrial copy number in resistant isolates further supports the observation of a fitness cost of resistance. These findings not only enhance our understanding of azole resistance in *A. fumigatus* but also pave the way for future investigations. Further exploration of these mechanisms could provide valuable insights into the development of novel therapeutic strategies to combat azole resistance in *A. fumigatus*.

## Methods

### Data acquisition

All data utilized in this study were derived from publicly accessible sequencing projects involving *A. fumigatus* isolates. In order to study strains resistant and sensitive to azole antifungals, public data were obtained from studies (Available from the project accession numbers: PRJEB27135, PRJDB10244, PRJEB8623, PRJNA638646, PRJNA798608, PRJNA592352, PRJNA548244, PRJNA961646, PRJNA592352) that provided descriptions about the resistance or susceptibility of the isolates through in vitro assays, totalling 318 whole genome sequences consisting of 148 azole resistant and 170 azole sensitive isolates. 32 of the resistant isolates are wildtype *cyp51A*, suggesting *cyp51A*-independent resistance mechanisms are present. The reads were downloaded from GenBank^47^ using SRA Toolkit v3.0.2^48^. The quality of sequences was verified by FastQC v.0.11.8 (Available at http://www.bioinformatics.babraham.ac.uk/projects/fastqc) and trimming of adapter sequences and quality filtering of the reads was completed with Trimmomatic v0.39^49^ using parameters “LEADING:20 TRAILING:20 MINLEN:60”.

### Assembly and annotation of nuclear genomes and mitogenomes

De novo genome assemblies were done using SPAdes v.3.14.0^50^. Genome completeness was inferred with BUSCO v5.4.4^51^ using *eurotiales_odb10* as the lineage parameter. For de novo assembly of mitochondrial sequences, the program GetOrganelle v1.7.6.1^52^ was used. After assembly, all mitochondrial circular sequences had their nucleotide positions reallocated to start from the same position using the fasta_shift script (available at https://github.com/b-brankovics/fasta_tools).

The genomes were annotated using automatic protein prediction with AUGUSTUS v3.5.0^53^ with the parameter ‘–species=aspergillus_fumigatus’. For predicting putative nuclear-encoded mitochondrial proteins, DeepLoc v2.0^54^ and Tppred v3.0^55^ were employed to identify signal peptides in proteins. Automatic annotation of mitogenome genes was performed using GeSeq v2.03^56^, followed by manual inspection. Mitogenome proteins were also annotated with MITOS v2.0^57^ and manually compared to the GeSeq results to generate a high-quality consensus set of annotated genes.

### Variant calling and GWAS

Processed reads were mapped to the reference genome of *A. fumigatus* Af293 (NCBI assembly code GCA_000002655.1) using Bowtie v2.3.5.1^58^. SAM files were converted to BAM format with SAMtools v1.15.1^59^. Single nucleotide polymorphisms (SNPs) were identified using the Genome Analysis Toolkit (GATK) v4.3.0.0^60^ module ‘HaplotypeCaller’, with an interval list of 468 genes predicted to encode mitochondrial proteins and an interval padding of 500. All variant files were combined using the ‘CombineGVCFs’ module to create a single file for genotyping with ‘GenotypeGVCFs’. Low-confidence SNPs were filtered using ‘VariantFiltration’ with the parameters “QualByDepth < 25.0 || MappingQuality < 55.0 || MappingQualityRankSumTest < −0.5 || FisherStrand > 5.0 || StrandOddsRatio > 2.5 || ReadPosRankSumTest < −2.0”, resulting in 3781 high-confidence SNPs. The final combined VCF file was converted to PHYLIP format using vcf2phylip v2.0 (Available in: https://zenodo.org/record/2540861) for phylogenetic analysis.

Genome-wide association (GWA) analysis was conducted to identify associations between SNPs and the resistance phenotype. From the 3781 variants in the VCF file, it was filtered out those present in less than 5% of samples. Principal components were extracted from these variants using TASSEL v5^61^, and the results were visualized using the R package ‘plotly’ (available at https://plot.ly). For the association analysis, the data were treated as binary—either resistant or susceptible—according to the descriptions of the original studies descriptions. We employed TASSEL v5^61^ to construct a kinship matrix to account for population structure and perform a linear mixed model (LMM) analysist. The *p*-values of associations were corrected using the false discovery rate (FDR) method, and significance was defined as *p* < 0.05. Finally, we created a Manhattan plot with *p*-value distribution using the qqman R package^62^ and the relationship of expected and observed *p*-values from the LMM analysis were visualized with a qqplot (Supplementary Fig. 1) made using the same package.

### Population structure based on mitochondrial protein encoding genes

The structure of *A. fumigatus* populations were based on the SNPs of nuclear-encoded mitochondrial proteins. Population structure analysis was performed with discriminant analysis of principal components (DAPC) using the adegenet R package v2.1.10^63^, in order to predict the number of populations and assignment of isolates. The likely number of populations was determined based on Bayesian information criterion (BIC) (Supplementary Fig. 4**)**, with k = 4 and 100 principal components retained.

### Comparative analyses

To compare the structure and visualize the differences between the regions of the mitogenomic sequences, synteny analysis was performed with Clinker v0.0.23^64^ using the GenBank files produced by GeSeq^56^. Pan-core analysis using protein sequences of mitochondrial genes was performed with the GET_HOMOLOGUES v3.5.4^65^. For phylogenetic analyses, high-confidence mitochondrial SNPs were used for the inference of the maximum likelihood tree, inferred with IQ-TREE v1.7^66^ using default parameters and the substitution model TVM + F + G4 automatically selected, and the ascertainment bias correction ( + ASC) added, which is recommended for SNP data. Generated trees were visualized in iTOL v6^67^.

F_st_ and Tajima’s D population genetic statistics based on the nuclear mitochondrial genes were calculated with VCFtools v0.1.16^68^ using a 10 kb non-overlapping window and plots were produced by the qqman R package^62^.

Proteins with evidence for mitochondrial transport signal peptides were used for NEM orthologs analysis with panOCT v3.23^69^; to calculate the openness of the ‘pan-genome’ (NEM orthologs) based on Heap’s law the R package ‘micropan’ was used^70^. The panOCT results were also used to compose tables to run in Scoary v1.6.16^28^, in conjunction with the SNP phylogeny previously produced, using the method described by Rhodes and colleagues^31^ to find associations of the NEM orthologs with the resistant and susceptible phenotypes.

The core and accessory NEM orthologs were assigned to KEGG orthology using BlastKOALA^27^ and classified according to the KEGG Brite hierarchical classification system.

The quantification of mitochondrial copy number was calculated by mapping the reads to each genome and mitogenome with Bowtie v2.3.5.1^58^ and calculating the average coverage with SAMtools v1.15.1^59^. The final result was obtained by dividing the mitochondrial DNA average coverage by autosomal DNA average coverage. For the analysis only genomes with the whole sequence average coverage above 50x were considered, totaling 126 isolates (17 Cyp51A independent resistants, 31 Cyp51A dependent resistants and 78 susceptible to azoles), in order to mitigate high deviations caused by low coverage genomes.

### Generation of *A. fumigatus* mutants

The replacement cassettes for the AFUA_8G01400 and AFUA_3G01290 genes were generated by in vivo recombination in *S. cerevisiae*, as previously described by Colot and colleagues^71^. Briefly, about 1.0 kb from each 5´UTR and 3´UTR flanking region of the targeted genes was PCR amplified from the *A. fumigatus* genomic DNA (CEA17 strain) using specific oligonucleotides: the 5´UTR sequence was amplified using the 5´UTR_gene_fw and 5´UTR_gene_rv primers and for 3´-UTR sequence amplification, the 3´UTR_gene_fw and 3´UTR_gene_rv primers were used. The primers 5´UTR_gene_fw and 3´UTR_gene_4_rv contained a short homologous sequence to the polylinker of the plasmid pRS426 and the 5´UTR_gene_rv and 3´UTR_gene_fw primers contained an overlapping sequence to the prototrophic marker pyrG, which was PCR amplified from the pCDA21 plasmid using the primers pyrG_fw and pyrG_rv. The individual gene fragments (5′ and 3′ UTRs and prototrophic marker gene) were transformed, together with plasmid pRS426, previously linearized with EcoRI and BamHI, into *S. cerevisiae* SC9721 using the lithium acetate method^72^. The genomic DNA of *S. cerevisiae* recombinant strains harboring the deletion cassettes was used as a template for PCR amplification utilizing TaKaRa Ex Taq™ DNA Polymerase (Clontech Takara Bio). Finally, the complete deletion cassette was used for *A. fumigatus* transformation. Southern blot analysis was performed to confirm the deletions (Supplementary Fig. 6). All primers used in this work are listed in the Supplementary Data 8.

### Minimum inhibitory concentrations (MIC) and minimum effective concentration (MEC)

The MIC and MEC were determined visually after incubation at 37 °C for 48 h following the guidelines outlined in the Clinical and Laboratory Standards Institute M38-A3 (CLSI. M38-A3 Reference Method for Broth Dilution Antifungal Susceptibility Testing of Filamentous Fungi Approved Standard). All chemicals used (itraconazole, voriconazole, caspofungin, isavuconazole, posaconazole and amphotericin B) were acquired from Sigma-Aldrich and assayed in a range from 4-0 µg/mL. In brief, the MIC and MEC assay were performed in RPMI-1640 or minimal medium (MM), using a 96-well flat-bottom polystyrene microplate from an initial inoculum of 2.5 × 10^4^/mL. Plates were incubated at 37 °C without shaking for 48 h, and growth inhibition was visually evaluated. The MICs for itraconazole, voriconazole, isavuconazole, posaconazole and amphotericin B were defined as the lowest concentration causing 100% growth inhibition compared to the drug-free growth control, determined visually after incubation at 37 °C for 48 h. The MEC for caspofungin was also assessed visually, using the same inoculum concentration after incubation at 37 °C for 24 and 48 h. The MEC was considered the lowest concentration, which led to the growth of small, compact, and rounded hyphae compared to positive growth. The *A. fumigatus* A1163 strain was used as a control in each MIC or MEC assay.

### Voriconazole persistence index

The persistence to voriconazole was analyzed by assessing the number of colonies forming unity (CFU). Briefly, a total 200 µL of MM containing 5×10^6^ CFU/mL conidia of each strain supplemented with voriconazole (4x MIC) was inoculated in 96-wells plate. After 96 h incubation at 37 °C, the wells were washed twice with sterile water and resuspended in 100uL of water. This volume was then plated in petri dishes containing solid MM (without drug). After 48 h incubation at 37 °C, the CFU was counted. The experiment was performed in at least 6 technical replicates and repeated 3 times independently. The viability of each strain was evaluated by plating 100 CFU of the fresh conidia in solid MM and monitoring of CFU number after 48 h incubation at 37 °C. The assay validation was done by first determining the azole persistence of A1160 parental strain and a previously described high persister strain, PDE-9^37^. The persistence index was calculated by:

$${\rm{Persistence\; index}}=\frac{{\rm{CFUs\; after\; azole\; exposure}}}{{\rm{Initial\; number\; of\; conidia}}}$$

## Supplementary information

Supplementary Information File

Supplementary Data 1

Supplementary Data 2

Supplementary Data 3

Supplementary Data 4

Supplementary Data 5

Supplementary Data 6

Supplementary Data 7

Supplementary Data 8

Supplementary Data 9

## Data Availability

The assembled nuclear and mitochondrial genomes were submitted to the NCBI’s under the BioProject ID PRJNA1152956.
