# Supplementary material for: Aspergillus fumigatus mitogenomes and their influence on azole-resistant and -susceptible populations
Source: NPJ Antimicrob Resist. 2025 Feb 27;3:15. doi: 10.1038/s44259-025-00083-6 (PMC11868573; doi:10.1038/s44259-025-00083-6)
Supplement: Supplementary file 1 — Supplementary Information File [file 44259_2025_83_MOESM1_ESM.pdf]

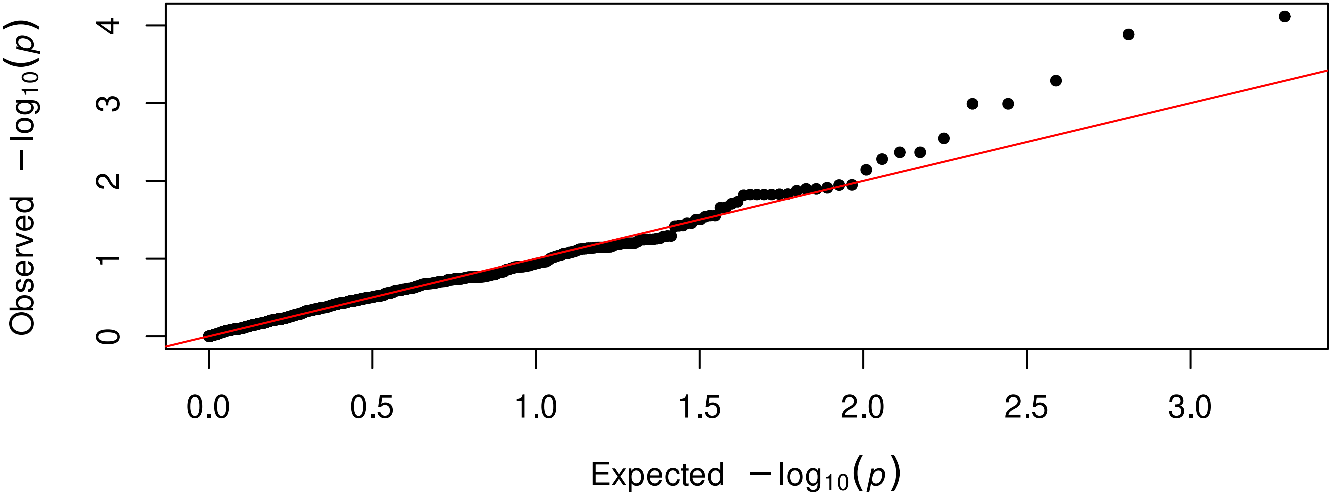

Supplementary Figure 1. QQplot comparing the expected and obtained p-values (Bonferroni correction) from the GWA analysis using a LMM for the SNPs of NEM proteins.

a.

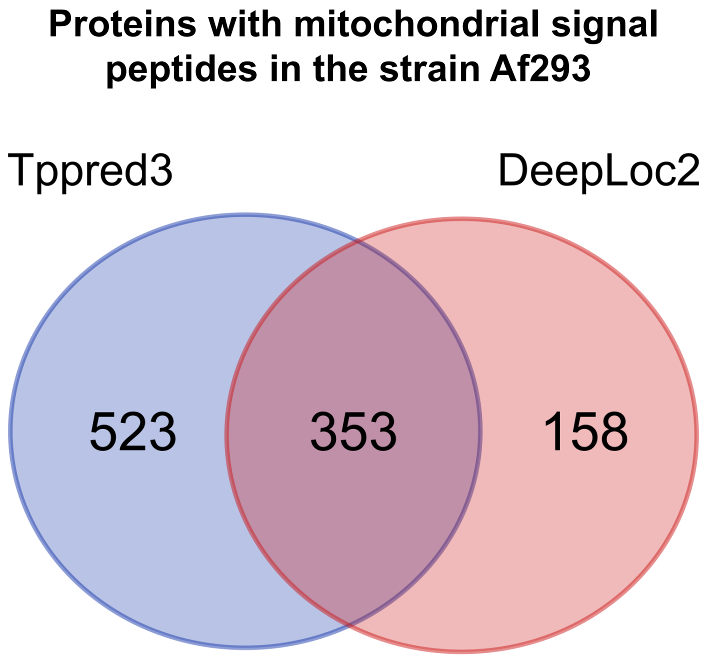

b.

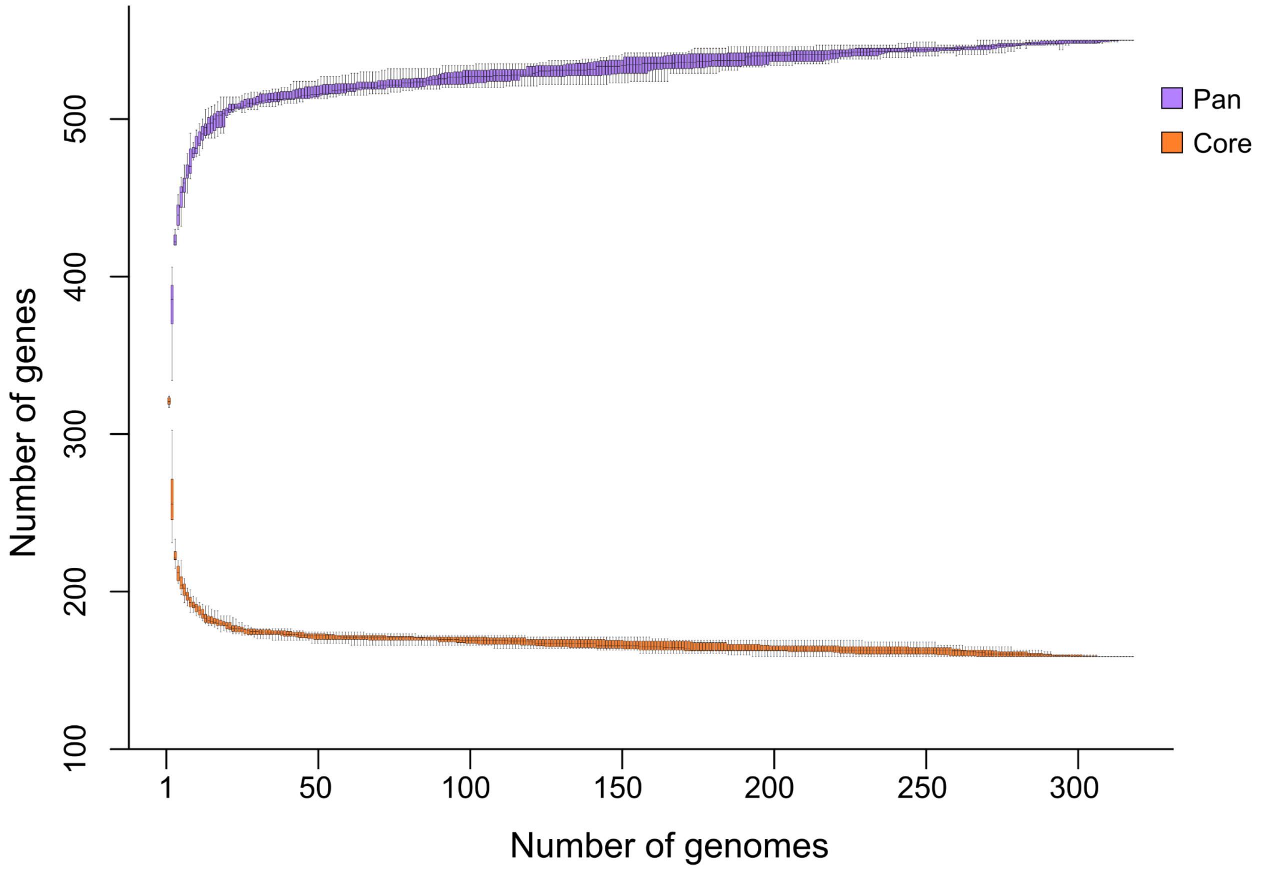

Supplementary Figure 2. Discriminant analysis of principal components (DAPC) biplot showing the distinct groups indicated by color. Clusters were determined based on Bayesian information criterion (BIC) with k = 4 and 100 principal components retained.

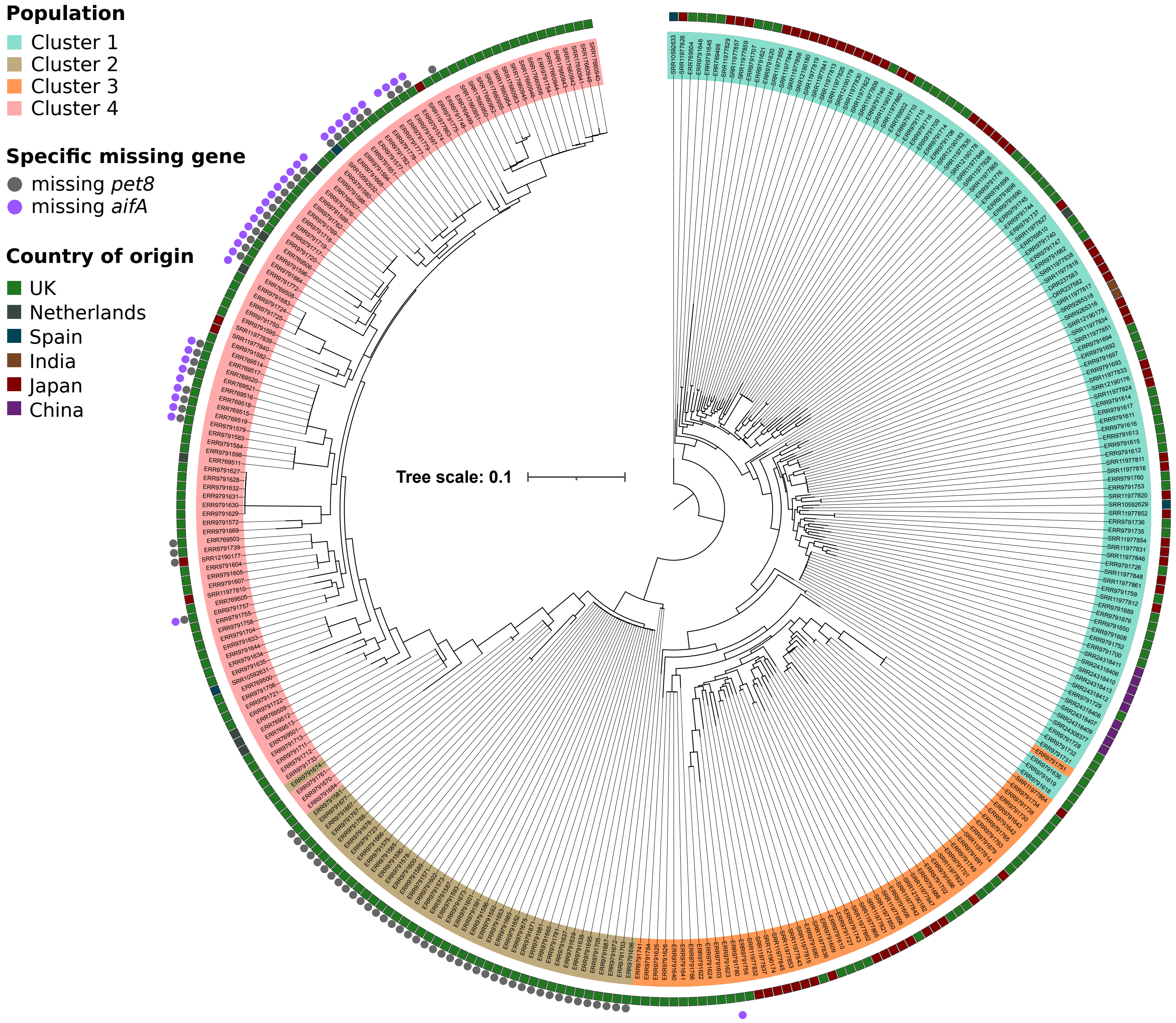

Supplementary Figure 3. Phylogenetic tree based on SNPs in nuclear encoded mitochondrial genes showing the subcluster distribution determined by DAPC analysis. Country of origin and cluster are indicated by a coloured box and the absence of pet8 and aifA is indicated by a coloured circle.

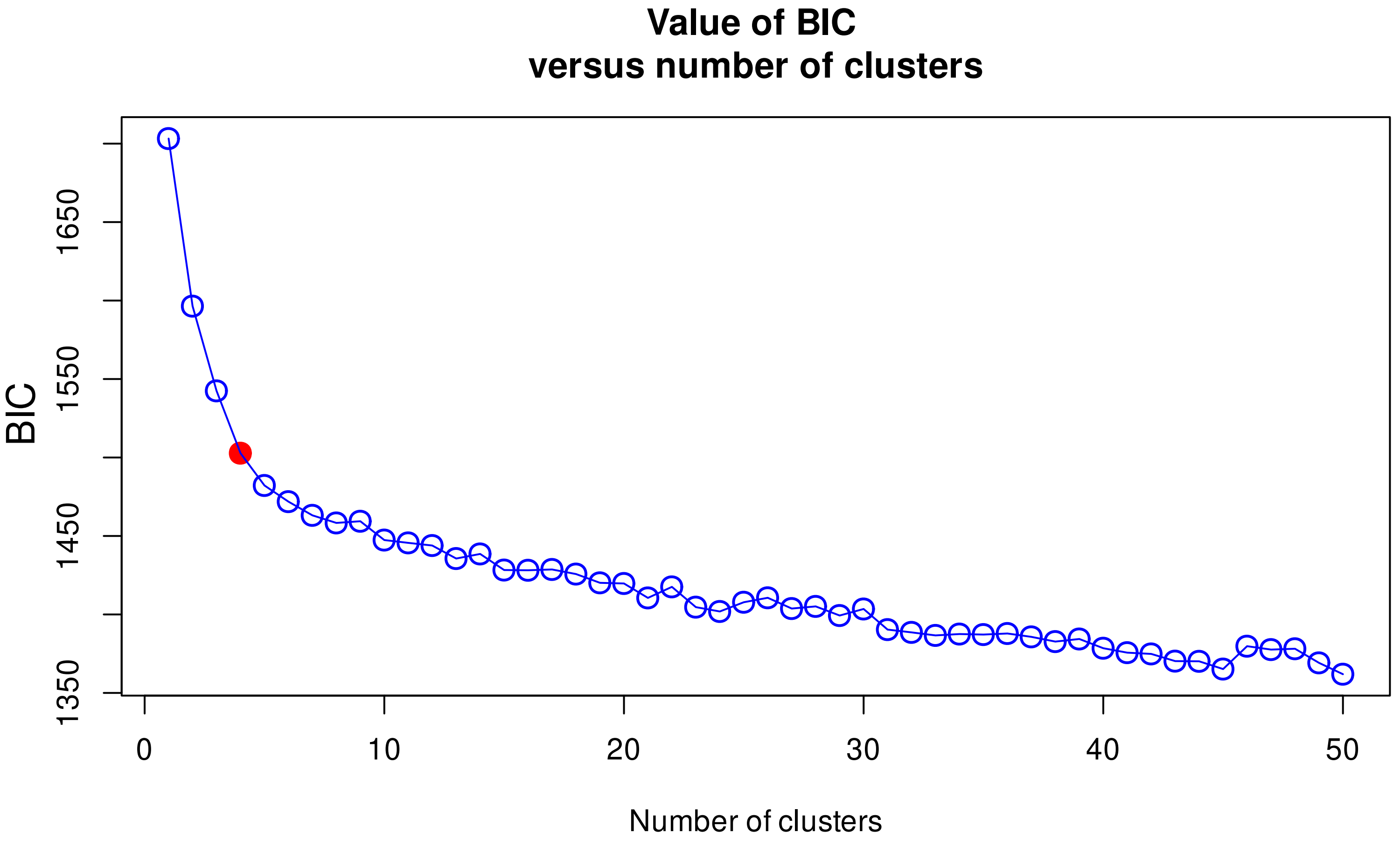

Supplementary Figure 4. Bayesian information criterion (BIC) values. The red circle represents k=4 chosen as the optimal number of populations.

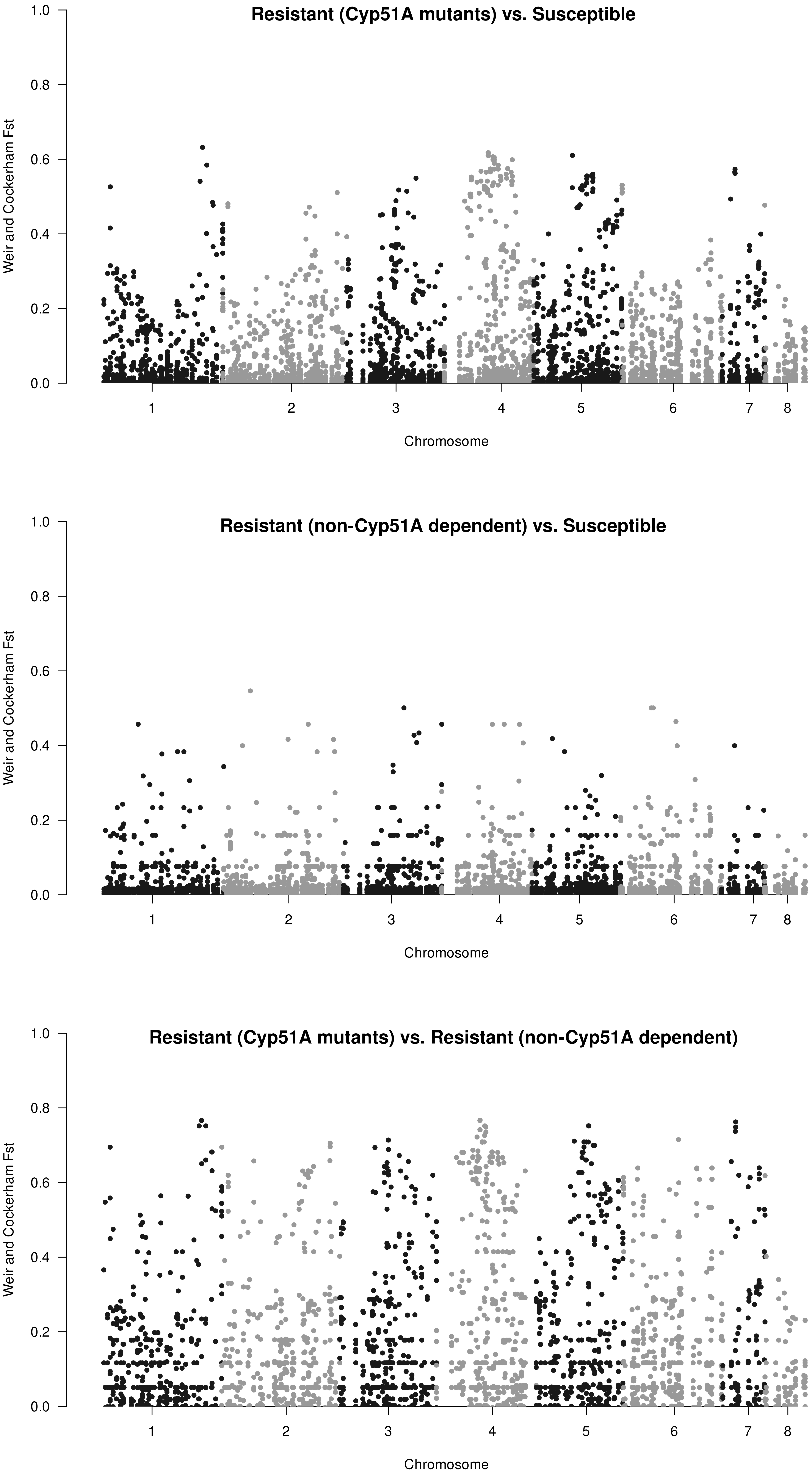

Supplementary Figure 5. Scatterplots of FST estimates for each chromosome. a. Resistant (cyp51A mutant) vs. Susceptible. b. Resistant (cyp51A independent) vs. Susceptible. c. Resistant (cyp51A mutant) vs. Resistant (cyp51A independent). Sliding 10 kb non-overlapping windows were used for FST calculation.

Figure 6. Validation of the deletion mutants. a. and b. Southern Blot schemes for *A. fumigatus* Δ*pet8* and Δ*aifA.*

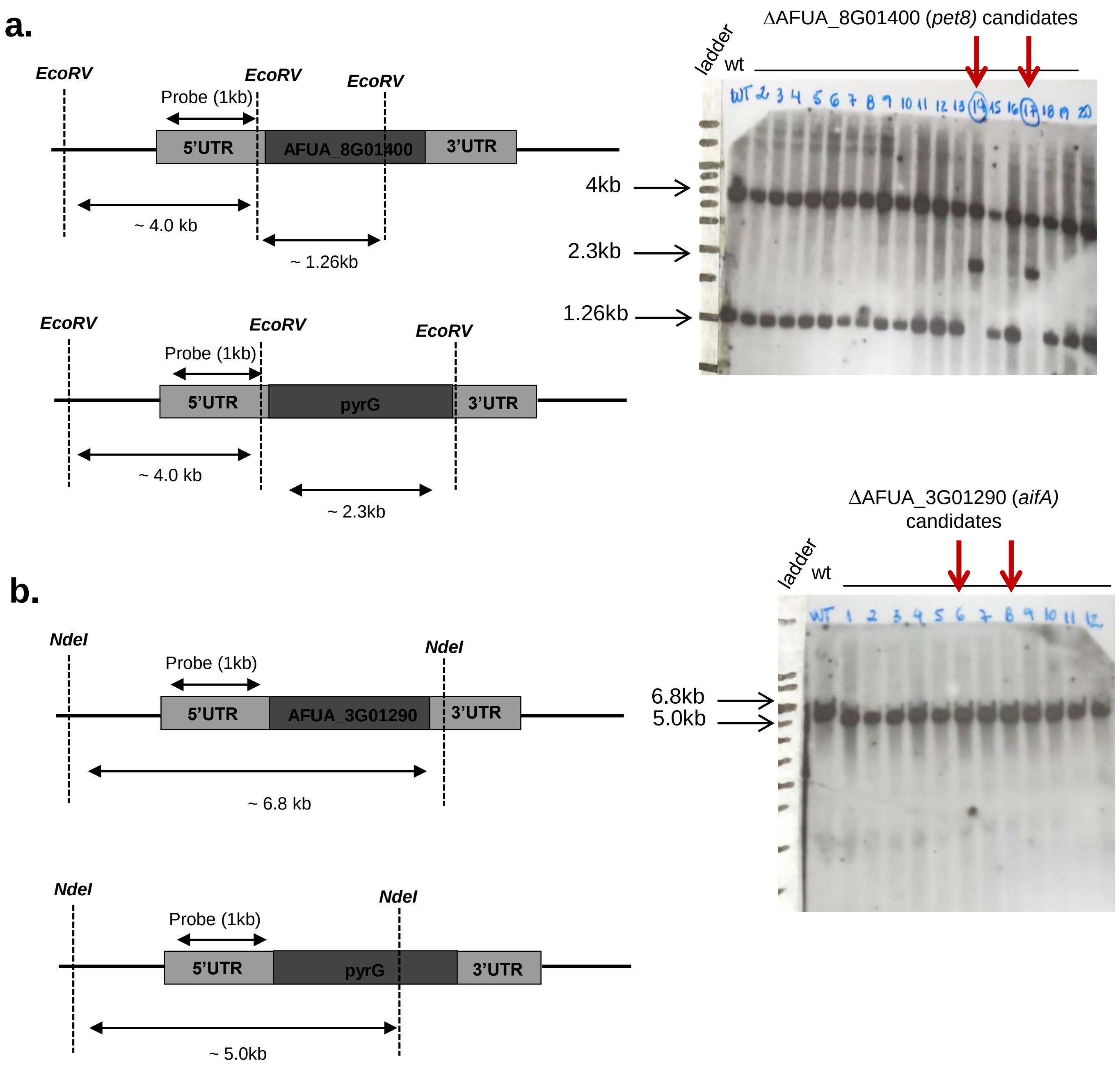

Figure 6. Validation of the deletion mutants. a. and b. Southern Blot schemes for *A. fumigatus* Δ*pet8* and Δ*aifA.* The red arrows indicate which deletion mutants were used in this work.
